# Time-to-reperfusion in patients with acute myocardial infarction and mortality in prehospital emergency care: meta-analysis

**DOI:** 10.1186/s12873-020-00356-5

**Published:** 2020-08-26

**Authors:** Xing Fu, Philip Wilson, Wing Sun Faith Chung

**Affiliations:** 1 Chengdu Center for Disease Control and Prevention, Chengdu, China; 2 University of Aberdeen, Aberdeen, The United Kingdom of Great Britain and Northern Ireland, Aberdeen, UK

**Keywords:** Acute myocardial infarction, Ambulance, Cardiovascular diseases, Emergency care, Prehospital time, Remote or rural, Rural health, Transit time

## Abstract

**Background:**

People living in rural areas usually suffer comparatively disadvantaged emergency health care than those living in urban areas, reasons including long transit time due to geographic factors. As for many time critical diseases, it is necessary to obtain treatment as quickly as possible.

**Methods:**

Screening of eligible studies were conducted based on inclusion an exclusion criteria. A comprehensive search was conducted by using following database: EMBASE, Medline, Cochrane library and Scopus. Quality assessment tool for observational cohort and cross-sectional study is used for assessing the risk of bias. The time group were defined based on the median or mean transit time among patients. In symptom onset-balloon time, we take 120 min transit time as the standard so patients in included studies are divided into two groups:less than 120 min (group A) and more than 120 min (group B). The collected data were used for quantitative analysis, they were inputted into Review Manager Software (v5.3) to produce summary results.

**Results:**

Ten studies representing 71,099 patients were included in the meta-analysis. All studies were retrospective and prospective observational studies and RCTs in which patients experienced ST-elevation myocardial infarction (STEMI) and were treated with percutaneous coronary intervention (PCI). Random effects meta-analysis of the point estimate was 0.69 (CI 0.60, 0.79). Heterogeneity between study results was evaluated via examination of the forest plots and quantified by using *I*^*2*^ statistic. Heterogeneity in two stage time was moderate among studies (*I*^2^ = 29%, *P* = 0.23).

**Conclusion:**

The meta-analysis for included studies report less mortality in less than 120 min symptom onset-balloon and door-balloon time than that in more than 120 min. It is necessary to optimize the prehospital system for rapid decision making and logical destination and mode of transport with prehospital notification of the cath lab so that the hospital is ready to optimize door to balloon time.

## Background

People in different geographic areas experiences discrepant in health services, which includes the density of paramedics, distances to treatment centers, and type of transportation to hospitals [1]. According to Horne et al. 2000, medical knowledge acquisition, demographic, symptom relevant, and clinical factors are likely to affect the prehospital transit time [2]. In many countries, it is common that people living in rural areas suffer comparatively disadvantaged emergency health care than those living in urban areas, most have experienced long transit time due to geographic factors [3]. Delays in receiving and reaching healthcare may lead to serious health issues [4]. Thus,it is vital to optimize the prehospital system so as to get to the hospital quickly.

As for many time critical diseases, it is necessary to obtain treatment as quickly as possible. In this context, acute myocardial infarction (AMI) serves as an example because it is a widespread disease all over the world, and it is one of the most serious type of coronary heart disease with a high mortality rate [4]. Minimizing the time of reperfusion therapy of AMI patients may be an effective method to control the death rate [5]. However, one of the main reasons influencing on therapeutic time may be distance. As so far, AMI mortality in rural areas is higher than that in urban areas because patients living in remote areas cannot receive suitable medical treatment in time [6]. Some individual factors also influence the timeliness of reception of AMI therapy, such as medically underserved setting and absence of medical knowledge [7]. Several studies have been conducted to identify the possibility of reducing mortality through reducing prehospital transit time. But a systematic review of the relationship between transit time and the mortality of acute myocardial infarction has not been conducted.

## Methods

### Aim

The aim of this study is to assess the relevant transit time and recovery of acute myocardial infarction in prehospital emergency care.

### Literature search strategy

We have systematically searched the following databases: EMBASE, Medline, Cochrane library and Scopus for all type of observational studies, qualitative studies and randomized controlled trials (RCTs). A combination of MeSH and keywords were involved in search strategy, including “transit time”, “acute myocardial infarction patients”, “emergency care”, “remote”. More details of the search processes were listed in Additional file 1. All included studies were managed by Refworks. Thirty- two studies were identified via screening article titles and abstracts for eligibility. Full- text articles were obtained to examine eligibility for data extraction. Any limitations in the studies were discussed among the three authors (FX, PW, WSFC). One author (FX) scanned all records first and then discussed any disagreements with other two reviewers (PW, WSFC).

### Eligibility criteria

Eligible studies were screened based on inclusion and exclusion criteria (Table 1). The studies were considered as eligible, if a) the study types were either an observational study, randomized controlled trial or a qualitative study. b) studies which report adult participants and acute myocardial infarction patients. c) trials were conducted in emergency department, hospital, prehospital setting and clinical setting. d) the outcomes were focused on measurable mortality. e) the article is from 1990 to present day and the article language is in English.

**Table 1:** Study inclusion and exclusion criteria

	Inclusion	Exclusion
Study type	Observational studies; Qualitative studies; RCTs.	Literature review; Unpublished report Conference
Participants	All gender; Acute myocardial infarctions patients; Adults	Non-AMI patients; Patients who are younger than 18 years old.
Setting	Emergency department; Hospital; Prehospital setting; Clinical setting	Home medicine
Aim and outcomes	Measurable mortality	No mortality record
Other	Reference is from 1990 to 2020; English	Pre 1990; Other language

### Assessment of risk bias

The nine selected studies were evaluated by a single researcher (FX) using Quality assessment tool for observational cohort and cross-sectional study (https://www.nhlbi.nih.gov/health-topics/study-quality-assessment-tools). One study was assessed by ROBINS-I (Risk Of Bias In Non-randomized Studies - of Interventions) [8]. The primary researcher (FX) then discussed the disagreements with another two reviewers (PW, WSFC). The risk of bias for observational studies was assessed by the following aspects: 1) Research question: Did the authors demonstrate their aim in research? Whether readers can get an idea of clear research goal easily. 2) Study population: Was selection of exposed and on-exposed group from the same population? Did the authors make inclusion and exclusion criteria based on demographics, medical history, and time period? Did the authors select participants according to inclusion criteria? If there are fewer than 50% of eligible people participated in the study, it should be considered an increased risk of bias. 3) Did the authors explain why select the number of participants to analyze? Whether they have recorded or discussed the statistical power of the study. 4) Whether exposure(s) of interest measured before the outcome(s) being measured, the step can determine whether or not an exposure causes an outcome. 5) Did the study allow adequate time to observe an outcome except for cross-sectional analyses? 6) For some exposures that are defined as a range, were different categories of exposure evaluated? Blind is unnecessary in some study types. 7) Were the exposures clearly defined and accurately measured? Were the measurement tools reliable? Were the results valid and measured objectively? 8) Repeated exposure assessment:were exposures are measured many times? The step can increase the accuracy of the results. 9) Can we be confident in the assessment of exposure? Was the outcome clearly defined and accurately measured? 10) Did the evaluators blind to the participants’ exposure status? 11) Was the follow up of participants adequate? A decreased follow-up rate may introduce bias and affect the outcome. 12) What are the key potential confounding variables for measurement and adjustment? Do they have an influence on the association between exposure(s) and outcome(s)?

### Groupings

Transit time was mostly classified into two-stage time groups, symptom onset-balloon time and door-balloon time. Onset-balloon time is defined as the time from the onset of symptoms to the first balloon inflation during percutaneous coronary intervention (PCI), and door-balloon time is defined as the time from arrival at the hospital door to the first balloon inflation during percutaneous coronary intervention [9].

The time group were defined based on the median or mean transit time among patients. In symptom onset-balloon time, we take 120 min transit time as the standard so patients in included studies are divided into two groups:less than 120 min (group A) and more than 120 min (group B). Some studies classified patients as two different transferred groups. For example, in Amit et al. 2006, population were grouped as patients with direct admission to PCI (median onset-balloon time is 210 min) and patients via emergency room to PCI (median onset-balloon time is 247 min). Both of their median time are more than 120 min so they are all classified to B group, otherwise, it is the A group. Similarly, we used the same way to set up time groups among door-balloon time. Several studies measured data from multiple time periods, therefore, these studies were pooled into two groups on the basis of median or mean.

### Data extraction

A data extraction form based on the Cochrane handbook for systematic review of intervention [10] was used by FX to collect information from included studies. The form contained: study ID (last name of the first author and publication date), study country, type of the study, transit time (min), the number of study population, number analyzed, mortality, age. We have reviewed articles in the publicly available journal, so ethics approval was not required.

### Meta-analysis

The collected data were used for quantitative analysis, they were inputted into Review Manager Software (v5.3) to produce summary results through comparing a dichotomous outcome. A forest plot was implemented through pooling data and comparing number of deaths in each group among individual studies. We used random effects models to calculate summary odds ratio and confidence interval of the summary estimate. Heterogeneity with *I*^2^ statistic was assessed. *I*^2^ values of ≤25, 50%, and ≥ 75%, correspond to small, moderate, and large amounts of heterogeneity respectively [11].

## Results

### Search results

**Fig. 1:**
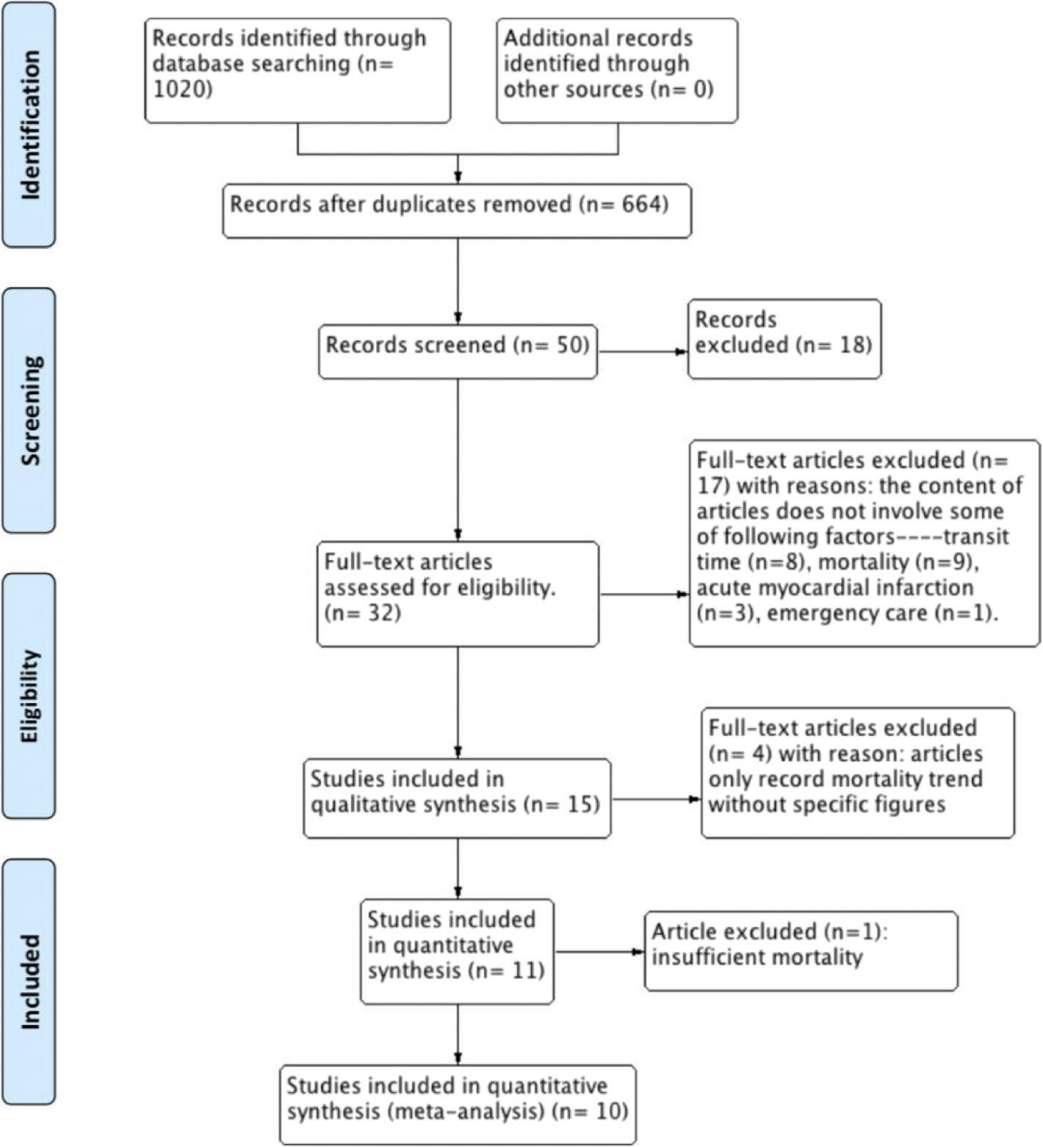
PRISMA flowchart [12]: outline of study screening process

The detailed process of article screening for the systematic review is shown in Fig. 1. A total number of 1020 publication were obtained. The remaining records were 664 after removing duplicates. Then 32 references were assessed after screening the title and abstract. Twenty-two records were excluded with the following reasons: eight studies lacked transit time data; nine did not report mortality; three did not represent acute myocardial infarction and one trial did not report patients in an emergency care setting. Another four articles were excluded because of the content did not show specific figures on mortality and one recorded insufficient mortality. Overall, ten studies were included for meta-analysis.

### Study characteristics

Ten retrospective and prospective observational studies and RCTs were included, in which it reported a total of 71,099 patients, whom experienced ST-elevation myocardial infarction (STEMI) and were treated with PCI. In a nutshell, three studies [9, 13, 14] were conducted in Israel, Korea and Japan respectively. The rest of the studies were carried out in America and Europe. The year of all studies were from year 2000 to 2012. The age of the reported participants was 62.2 ± 15 (mean ± SD) years old. The majority of results showed a decreased risk of mortality in shorter transit time compared to the longer transit time. Participants in Cannon et al. 2000 were divided into two groups based on the median time (Onset- balloon time was 234 min; door-balloon time was 116 min) and population from Cho et al. 2011 were divided according to the mean time (330 min). We have used the reported 30-day mortality or in-hospital mortality as the primary result for this systematic report. Included studies involved each group associated transit time (median or mean) and 30-day mortality. Brodie et al. 2003 and Cho et al. 2011 only recorded onset-balloon time. Brodie et al. 2006 and McNamara et al. 2006 only measured door-balloon time and six trials included both of two-stage time. More details are listed in Table 2 below.

**Table 2:**
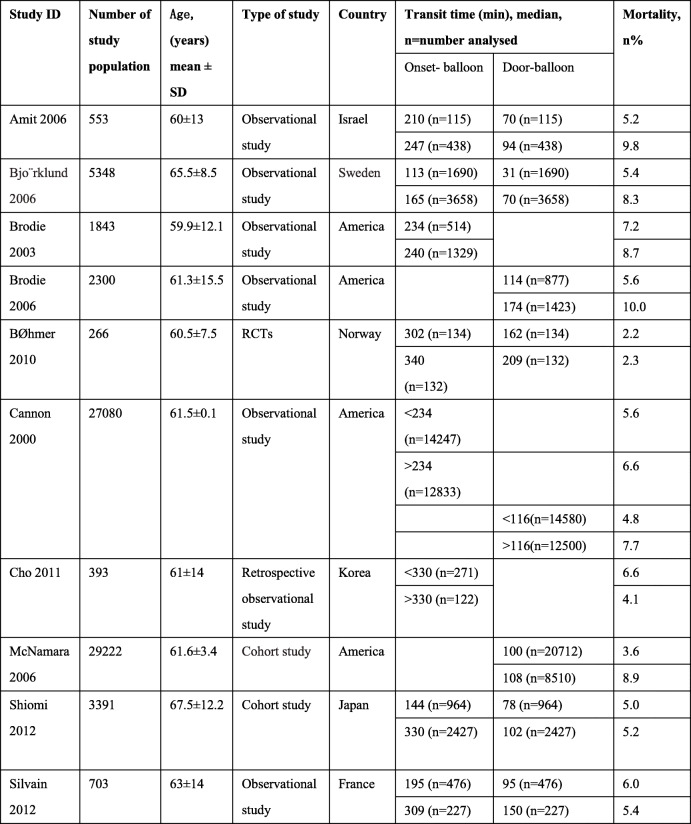
Included study characteristics

### Comparison of transit time in different studies and grouping results

**Fig. 2:**
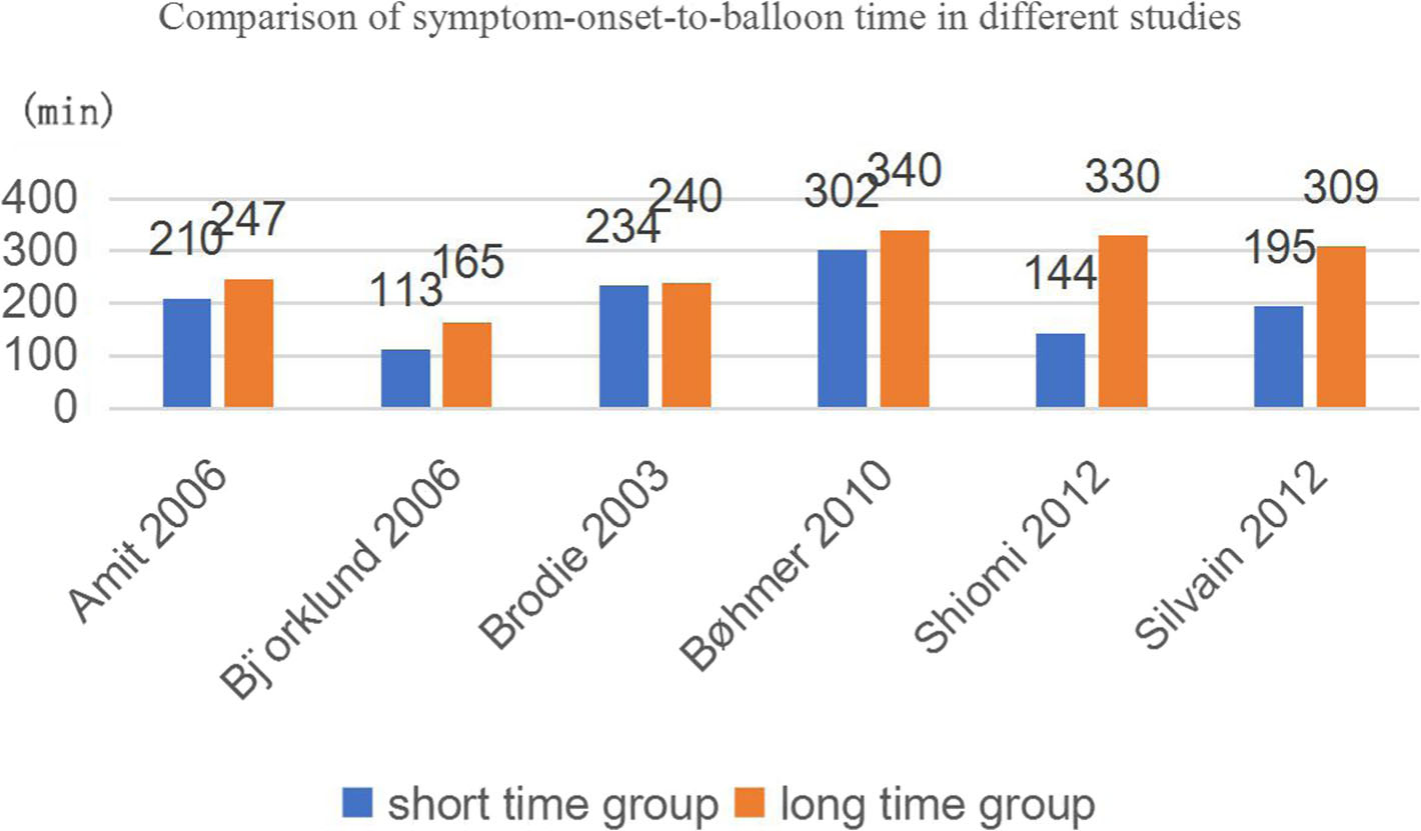
comparison of symptom-onset-to-balloon time in different studies

The median symptom-onset-to-balloon time six studies are shown in Fig. 2. Cannon 2000 was excluded from the histogram because of the absence of median time in each group. Cho 2011 was also excluded as it only recorded mean time of all patients. It can be seen that the patients from Bjo¨rklund 2006 may have the shortest time for first balloon inflation, with 113 min of median time and 165 min, while patients from BØhmer 2010 were likely to have the longest time to first balloon inflation, 302 min and 340 min. As for other groups, the median symptom-onset-to-balloon time was 210 min and 247 min Amit 2006 respectively. Brodie 2003 reported extremely similar time data, 234 min and 240 min. There was a larger gap between the two groups in Shiomi 2012, with 144 min and 330 min respectively. 195 min and 309 min were recorded in Silvain 2012. Therefore, only patients who experienced 113 min of median time are involved in group A,number analysed is 1690. The rest (10414) are put in group B.

**Fig. 3:**
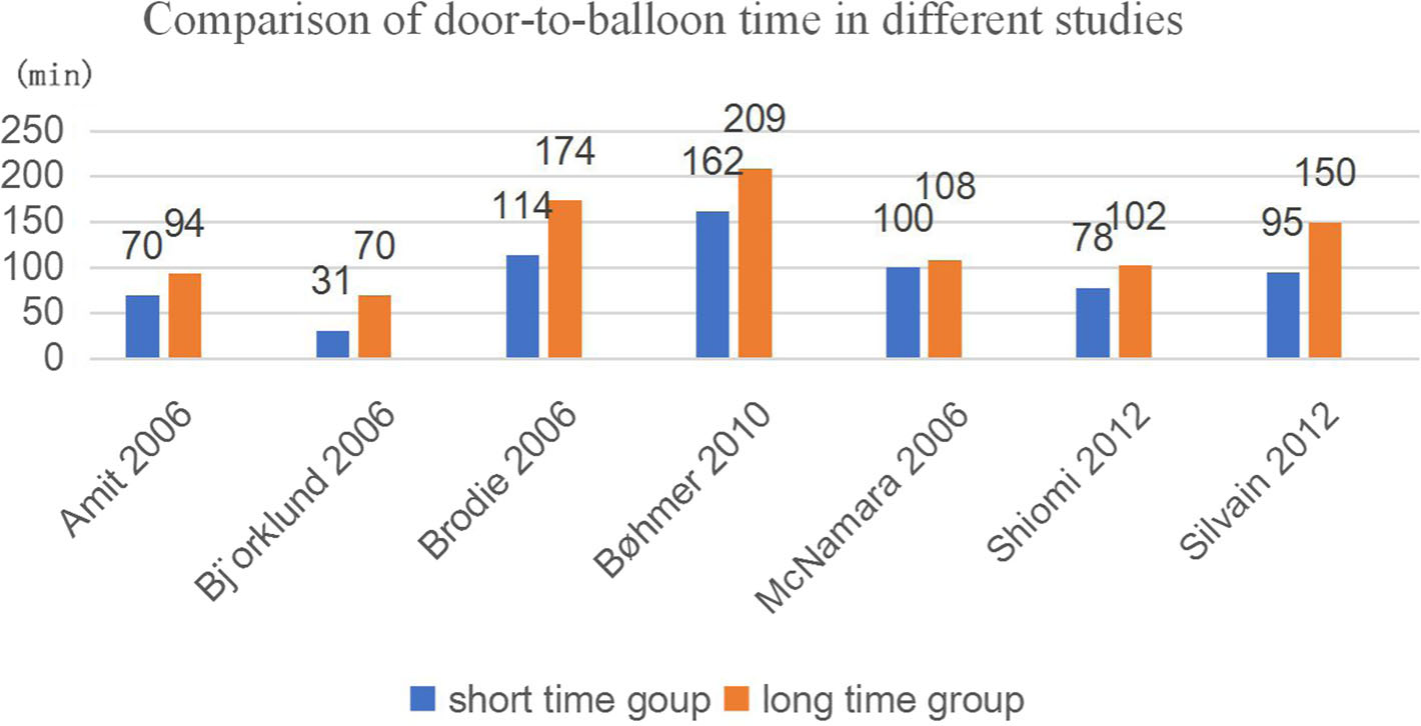
comparison of symptom-onset-to-balloon time in different studies

The median door-balloon time in seven studies are shown in Fig. 3. Bjo¨rklund 2006 presented the least median time for patients from arrival at the hospital to first balloon inflation, along with 31 min and 70 min, while BØhmer 2010 reported the most median time, 162 min and 209 min respectively. Patients from Brodie 2006 were also experienced longer transit time, 114 min and 174 min of two groups. There were 70 min and 94 min of two groups in Amit 2006. McNamara 2006 showed similar periods time in its two groups, 100 min and 108 min in two groups. There are 39,867 patients in group A and 1916 patients in group B.

According to the Table 2, there are 91 deaths in group A and 727 deaths in group B in symptom onset-balloon time. In door-balloon time 2199 patients of group A and 160 patients of group B died respectively.

### Symptom onset-balloon time and door-balloon time

**Fig. 4:**
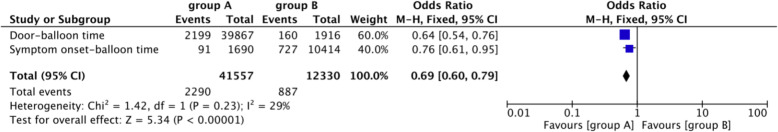
forest plot for number of deaths for symptoms onset-balloon time (Revman)

Relative odds ratio and corresponding 95% confidence interval are showed in Fig. 4. Random effects meta-analysis of the point estimate was 0.69 (CI 0.60, 0.79). Heterogeneity between study results was evaluated via examination of the forest plots and quantified by using *I*^*2*^ statistic. Heterogeneity in two stage time was moderate among studies (*I*^2^ = 29%, *P* = 0.23).

## Discussion

The estimates from meta-analysis for overall number of patients and the number of deaths within 30 days reported an effect size of 0.69 (CI 0.60, 0.79) for two stage time. There is an evidence suggesting that mortality occurred less frequently in the less than 120 min of transit time group than in the more than 120 min of transit time group (ratio < 1) according to the results of forest plot, although there was no significant difference between transit time (both symptom-onset-to-balloon time and door-balloon time) and short-term mortality. Moreover, there is a comparatively large difference in the transit time of each trial from included studies. However, Brodie 2003 reported extremely similar median time, 234 min and 240 min of short and long time group. One possible explanation may be that a large difference of sample size lead to abnormality of the outcome. There are 1705 patients from short time group, while only 138 patients in long time group. Meanwhile, patients in two groups have different clinical characteristics. Population in short time group were non-shock patients, whereas AMI patients in long time group suffered shock. It is possible that shock patients may experience longer emergency care time on the spot [15]. The bias introduced by follow-up was not considered as an assessment of mortality was conducted within a short-term (30-day).

Deficiencies often existed in studies. The studies involved in this review include different demographic characteristics. Emergency medical services in low- and middle-income countries (like East Asia and the Pacific, Latin America and the Caribbean, South Asia, and Sub-Saharan Africa)are more likely to result in a disadvantaged recovery. Patients’ rescue time might be delayed because of insufficient ambulance, lacking appropriate prehospital care and necessary supplies [16].

Additionally, screening trials include retrospective and prospective observational studies, recall bias is less likely to be avoided in retrospective studies and survivor bias could affect clinical outcomes [9]. It is difficult that to assess the time of onset of symptom sometimes so that it would result in errors in door-balloon time [17]. Different trials that were grouped with different standards and the results could be influenced by variables. Clinical characteristics of patients would introduce confounders into differences of mortality of different periods of time so that confounder bias caused by underlying disease is likely to be ineluctable. For example, cardiogenic shock often occurs in myocardial infarction patients,which can result in a significant in-hospital mortality. in Brodie 2003, patients with shock experienced longer time- to-reperfusion and mortality are also different from patients without shock. Therefore, some patients would suffer higher mortality even if there is less than 120 min symptom onset-balloon time. A few studies [14, 18] showed the opposite result among mortality with other studies, which was caused possibly by insufficient sample size.

In addition to characteristics mentioned above, a number of others vary in different studies, which can lead to heterogeneity. These situations include differences in inclusion and exclusion criteria, the instruments used and the medical levels of paramedical personnel [16].

### Limitation

Most included studies reported 30-day mortality so we only use the data, which might involve in many confounders. Not all studies had reported median or mean of symptom onset-balloon time and door-balloon time and corresponding mortality directly. Some studies had only recorded the approximate range of the transfer time and the mortality in the time range. Besides, participation in these studies from observational study was not randomised. We limited the search language to English, which would contribute to linguistic bias. Meanwhile, limit number of studies may not be representative.

### Comparison with previous studies

This review showed patients with STEMI are more likely to survival when experiencing shorter transit time, in spite of other variables. Needleman et al. 2011 reported that the availability of skilled staff, staffing levels and the number of paramedics are potential factors to contribute to mortality [19]. Kulkarni et al. 2013 showed a similar outcome. 30-day mortality rate of patients with acute myocardial infarction in the low density of cardiovascular disease experts was higher than those in high-density areas, which suggested that the outcome of patients with AMI might be influenced by the availability of cardiology specialists in regional care systems [20]. Other factors, including patients’ own condition (sleep deprivation and fatigue [21], genetic factor) and distance from home to hospital [22], can result in increased risk of death for AMI.

As for the factors affecting the transit time, availability of cardiologists and cardiac catheterization laboratory staff are considered to be associated with that [23]. The increase in the time interval between getting the electrocardiogram (EGG) and arriving at the catheterization laboratory would lead to the increase in almost all door-balloon time [23]. Delay to make decision to seek care and delay to receive care may be the reason for the increase in the overall transit time [2, 4].

## Conclusion

The meta-analysis for included studies report less mortality in less than 120 min symptom onset-balloon and door-balloon time than that in more than 120 min. Multiple factors, such as availability of skilled staff, the density of paramedics and necessary supplies could lead to differences in mortality and time intervals. It is necessary to optimize the prehospital system for rapid decision making and logical destination and mode of transport with prehospital notification of the cath lab so that the hospital is ready to optimize door to balloon time. There may be some measures to optimize the prehospital system to improve recovery. Ambulance stations would be set according to incidence of AMI. More ambulance stations could be set up in areas with higher incidence rate to shorten transit time as soon as possible. Besides, prehospital care should contain the process with from scene of symptom onset to hospital at least, which requires that emergency personnel should possess the ability of evaluating patients’ condition and providing first aid. In addition, Development of severe heart failure could be prevented by early reperfusion therapy, which provided a possibility with application of thrombolytics in transit to improve the consequences [24]. Indeed, it would be challenged for paramedics to implement these complicated treatment in transit process. In future, employing more interested community volunteers could be a feasible method. They would be trained to have some basic rescue skill, including cardio-pulmonary resuscitation and transport of patients.

## Supplementary information

**Additional file 1.**

## Data Availability

The datasets used and/or analysed during the current study are available from the corresponding author on reasonable request.

## Abbreviations

STEMI: ST-elevation myocardial infarction
PCI: Percutaneous coronary intervention
AMI: Acute myocardial infarction
EGG: Electrocardiogram
