# Supplementary material for: Time-to-reperfusion in patients with acute myocardial infarction and mortality in prehospital emergency care: meta-analysis
Source: BMC Emerg Med. 2020 Aug 26;20:65. doi: 10.1186/s12873-020-00356-5 (PMC7448494; doi:10.1186/s12873-020-00356-5)
Supplement: Supplementary file 1 — Additional file 1. [file 12873_2020_356_MOESM1_ESM.pdf]

Appendix A: search strategy

Database	Concept			
	Rural/remote	Time	Acute myocardial infarction	Emergency
Scopus	1.ALL ("rural health" OR "RURAL POPULATION" OR "RURAL nursing" OR "RURAL health services")<br>2. ALL (remot* OR rural*)	1. ALL ("transit* tim*" OR "tim* transit*")<br>2. ALL ("transport* tim*" OR "tim*transport*")<br>3. ALL ("tim* to hospital*")<br>4. ALL ("tim* to treat*" OR "treat* tim*")<br>5. ALL ("prehospital* time*" OR "or pre-hospital*time*")<br>6. ALL ("deliver*tim*")<br>7. ALL ("time factors")<br>8. ALL ("tim* element*")<br>9. ALL ("tim*critical*")	ALL ("Cardiovascular Diseases" OR "Coronary Disease" OR "Coronary Artery Disease" OR "Myocardial Infarction")	1.ALL (ambulance OR "emergency health service" OR "paramedical personnel" OR "rescue personnel" OR "emergency care")<br>2.ALL (ambulanc* OR par amedic* OR para-medic*)
Medline/Embase	1.exp RURAL HEALTH/ or RURAL POPULATION/ or exp HOSPITALS, RURAL/ or exp RURAL NURSING/ or exp RURAL HEALTH SERVICES/<br>2. (remot* or rural*).af.	1. (transit* tim* or tim* transit*).af.<br>2. (transport* tim* or tim* transport*).af.<br>3. tim* to hospital*.af.<br>4. (tim* to treat* or treat* tim*).af.<br>5. prehospital* time* or pre-hospital*time*).af.<br>6. deliver*time*.af.<br>7. exp Time Factors/<br>8. exp TIME/<br>9. tim* element*.af.<br>10. time critical*.af.	1.exp Myocardial Infarction/<br>2.exp Cardiovascular Diseases/ or exp Coronary Disease/ or exp Coronary Artery Disease/	1. exp ambulance/<br>2. exp emergency health service/ or exp paramedical personnel/ or exp rescue personnel/<br>3. exp emergency care/<br>4. (ambulanc* or paramedic* or para-medic*).af.
Cochrane library	1. MeSH descriptor: [Rural Health] explode all trees<br>2. RURAL POPUL* (Word variations have been searched)<br>3. RURAL nur*<br>4. RURAL health service*<br>5. health service* rural*<br>6. remot* or rural* (Word variations have been searched)	1."tim* transit*" or "transit* tim*"<br>2."time to hospital*" or "time to admiss*"<br>3."transport* tim*" or "tim* transport*"<br>4."tim* element*" or "element* tim*"<br>5."treat* tim*" or "tim* treat*"<br>6."tim* critic*" or "crit* tim*"<br>7."prehospital* Tim*" or "pre-hospital* tim*"<br>8. "deliver* tim*"<br>9. MeSH descriptor: [Time Factors]<br>10. MeSH descriptor: [Time]	1.MeSH descriptor: [Myocardial Infarction]<br>2.MeSH descriptor: [Cardiovascular Diseases]<br>3.MeSH descriptor: [Coronary Disease]<br>4.MeSH descriptor: [Coronary Artery Disease]	1.MeSH descriptor: [Ambulances]<br>2.MeSH descriptor: [Emergency Medical Services]<br>3.MeSH descriptor: [Allied Health Personnel]<br>4. ambulanc* or paramedic* or para-medic*
